# *Orientia tsutsugamushi* and *Epstein-Barr Virus* coinfection presenting with transient fluctuating hearing loss: a case report

**DOI:** 10.3389/fimmu.2026.1750100

**Published:** 2026-03-27

**Authors:** Yibo Zhai, Miao Yu, Liniu Cheng, Xinsheng Liu, Junqiang Yan

**Affiliations:** 1 Department of Neurology, The First Affiliated Hospital, College of Clinical Medicine of Henan University of Science and Technology, Luoyang, China; 2 Key Laboratory of Neuromolecular Biology, The First Affiliated Hospital, College of Clinical Medicine of Henan University of Science and Technology, Luoyang, China

**Keywords:** coinfection, *Epstein-Barr virus*, hearing loss, scrub typhus, *Orientia tsutsugamushi*, metagenomic next-generation sequencing

## Abstract

Scrub typhus, caused by the obligate intracellular bacterium *Orientia tsutsugamushi*(*O. tsutsugamushi*), is an acute febrile illness. While neurological complications are known, hearing loss is an uncommon manifestation, and coinfection with *Epstein-Barr virus*(EBV) presents unique diagnostic and pathophysiological challenges. A 58-year-old woman presented with a 5-day history of high fever, severe headache, and constitutional symptoms. She reported transient, fluctuating bilateral hearing loss. Examination revealed characteristic eschars on her legs. Laboratory findings indicated hepatic impairment and systemic inflammation. Metagenomic next-generation sequencing (mNGS) of cerebrospinal fluid detected *O. tsutsugamushi* and EBV. EBV serology profile (VCA-IgG+, VCA-IgM-, EBNA-IgG+) suggested viral reactivation. The patient failed to respond to initial beta-lactam antibiotic therapy but showed rapid and complete resolution of symptoms, including hearing loss, after initiation of doxycycline. At the 1-month and 3-month follow-up, audiological assessment confirmed normal hearing. This case highlights a rare presentation of scrub typhus with EBV coinfection involving fluctuating hearing loss. The dramatic response to doxycycline suggests this auditory symptom may be a reversible, immune-mediated complication of *O. tsutsugamushi* infection. Physicians should be aware of this potential manifestation in endemic areas. The immunological interplay between these pathogens warrants further investigation.

## Introduction

Scrub typhus is a significant vector-borne zoonosis caused by *Orientia tsutsugamushi*(*O. tsutsugamushi*), with an estimated one billion people at risk globally. The classic clinical triad includes fever, rash, and an eschar at the site of the chigger bite. Neurological manifestations, including meningitis and encephalitis, occur in a subset of patients (1). Sensorineural hearing loss has been documented but is an unusual presenting feature.

The pathophysiology of scrub typhus involves infection of vascular endothelial cells, which can lead to vasculitis and perivascular inflammation in various organs (2), potentially including the inner ear. *O. tsutsugamushi* is intrinsically resistant to beta-lactam antibiotics, making early and accurate diagnosis critical for initiating effective treatment, typically with doxycycline or azithromycin.

*Epstein-Barr virus*(EBV) is a ubiquitous herpesvirus that establishes lifelong latency. Reactivation can occur during periods of physiological stress or immunosuppression (3). Coinfections with *O. tsutsugamushi* and viruses like EBV are poorly understood but may modulate the host immune response and disease presentation.

We present a case of *O. tsutsugamushi* and EBV coinfection characterized by transient fluctuating hearing loss, an uncommon symptom that resolved completely with appropriate antibacterial therapy. This case expands the clinical spectrum of scrub typhus and underscores the value of advanced diagnostic tools like metagenomic next-generation sequencing(mNGS).

## Case presentation

### Patient information and clinical history

**Figure 1:**
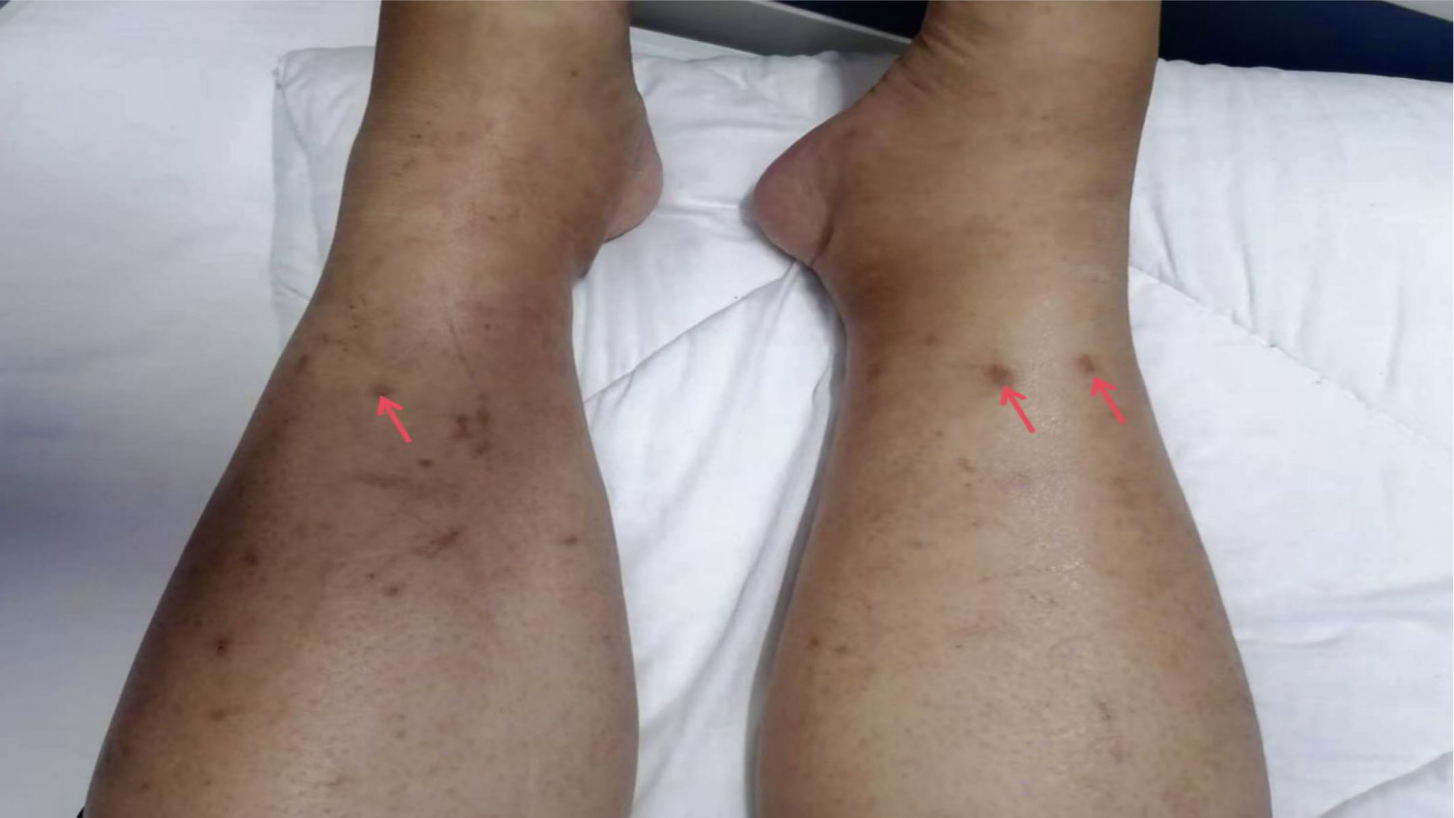
Multiple characteristic skin lesions on thelower segments of both calves, witheschars mainly distributed in the anteriortibial region, totaling three (one on the leftcalf and two on the right calf). The skinlesions were round or oval, approximately 1–2 millimeters in diameter. The center ofthe eschar was black or dark brown, dry, hard, and slightly raised, with a clearboundary from the surrounding normaltissue. A red halo about 2–3 millimeters wide surrounded the eschar, with nosignificant edema or fluctuation in thelocal skin.

A 58-year-old female farmer with pre-existing hypertension, type 2 diabetes, and coronary artery disease presented to our institution in August 2025 with a five-day history of high-grade fever (peak temperature 39.2 °C) and severe, persistent frontal headache. The patient resided in a rural area with significant arthropod exposure but denied recent travel to recognized endemic regions. Her symptoms included chills, chest tightness, urinary urgency with dysuria, and diarrhea (3–4 episodes daily). Notably, she described fluctuating bilateral hearing loss characterized by sudden auditory deterioration requiring loud verbal repetition, occasionally accompanied by auditory hallucinations. The patient reported no improvement despite three days of outpatient antibiotic therapy preceding admission. Her medication history included antihypertensives and oral hypoglycemics, with no known drug allergies. Physical examination revealed multiple characteristic skin lesions on the lower segments of both calves (**Figure 1**).

### Physical examination and clinical findings

On admission, the patient was febrile (38.9°C) with stable hemodynamic parameters. Dermatological examination identified three eschars on her lower legs measuring 3–5 mm in diameter, with central black/brown crusts surrounded by erythematous halos. Due to the subacute disease course, these lesions were partially healed, with characteristics documented from both physical examination and patient history. Neurological examination confirmed bilateral hearing impairment but was otherwise unremarkable, with no meningismus, focal deficits, or cranial neuropathies. Laboratory investigations revealed elevated liver enzymes (ALT 118 U/L, AST 230 U/L), hypoalbuminemia (35.4 g/L), markedly elevated inflammatory markers (C−reactive protein: 115.92 mg/L), and coagulation abnormalities (elevated D−dimer: 9.75 mg/L). Lumbar puncture demonstrated mild pleocytosis (WBC 0.028 × 10^9^/L) with normal protein and glucose levels (**Table 1**). Serological tests for other common pathogens and autoimmune conditions were negative.

**Table 1:** Key laboratory parameters at admission.

Parameter	Value	Reference range
Liver Function		
ALT (U/L)	118	0-32
AST(U/L)	230	14-36
Albumin(g/L)	35.4	40-55
Inflammatory Markers		
IL-6(pg/mL)	16.18	0-7
hs-CRP(mg/L)	115.92	0-5
Coagulation Profile		
D-dimer(mg/L)	9.75	0-1.0
Electrolytes		
Sodium(mmol/L)	133.9	137-147
Lumbar puncture		
WBC-BF (10^9^)	0.028	0-0.01

### Diagnostic assessment

**Figure 2:**
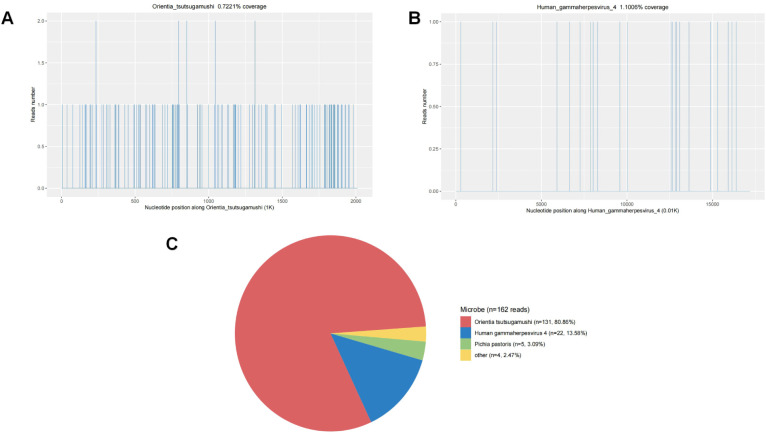
**(A, B)** A visual chart showing the proportionof each species’nucleic acid genomicsequences identified in the sample relativeto the total effective sequencing data. **(C)** Additionally, a chart that statistics theproportion of each species in the sample.

The diagnostic evaluation employed a systematic approach to address the complex presentation. Imaging studies included cranial CT demonstrating multiple cerebral infarctions and white matter degeneration, with MRI confirming bilateral white matter hyperintensities (Fazekas grade 2) and cerebral arteriosclerosis (**Table 2**). Chest radiography and abdominal ultrasonography were unremarkable. Differential diagnosis included scrub typhus, leptospirosis, murine typhus, viral encephalitis, and autoimmune disorder. Diagnostic confirmation was obtained through metagenomic next−generation sequencing (mNGS) of cerebrospinal fluid using the Salus Pro platform, generating approximately 38 million single-end 76 bp reads (total data ~37.6 MB), which identified *O. tsutsugamushi*(sequence count: 131) and *Human gammaherpesvirus 4 *(*Epstein-Barr virus*) (**Figures 2A-C**). EBV serological testing showed positive VCA-IgG, negative VCA-IgM, and positive EBNA-IgG, a profile highly suggestive of past infection with subsequent reactivation. The final diagnosis confirmed through comprehensive laboratory and molecular testing.

**Table 2:** Imaging results.

Testitem	Result
Cranial CT	Multiple cerebral infarction, white matter degeneration.
Cranial MRI	1.Bilateral cerebral hemisphere white matter hyperintensity (presumed vascular), Fazekas grade 2; 2. Multiple vascular-derived lacunars in the brain; 3. Cerebral arteriosclerosis.
CT of the upper abdomen	Thickening of the gallbladder wall; The spleen is slightly larger, and the side spleen is subspleen.
Chest CT	Slight exudation from both lungs; Multiple ground-glass and solid nodules in both lungs; multiple calcified nodules in both lungs; The left atrium is slightly enlarged, the pulmonary artery trunk is slightly widened, the left coronary artery is hardened, and a small amount of pericardial fluid accumulates; Effusion in the left pleural cavity and thickening of the pleura on the right.
Chest CT	Slight exudation from both lungs; Multiple ground-glass and solid nodules in both lungs; multiple calcified nodules in both lungs; The left atrium is slightly enlarged, the pulmonary artery trunk is slightly widened, the left coronary artery is hardened, and a small amount of pericardial fluid accumulates; Effusion in the left pleural cavity and thickening of the pleura on the right.

### Therapeutic intervention and outcome

Initial empirical antibiotic therapy with intravenous cefoperazone-sulbactam (3g every 8 hours) was initiated upon admission for broad-spectrum coverage. However, after 72 hours of treatment, the patient’s febrile symptoms persisted with no clinical improvement. Upon receipt of the mNGS results identifying Orientia tsutsugamushi, the therapeutic regimen was modified based on the pathogen’s intrinsic resistance to beta-lactam antibiotics. Targeted therapy was promptly initiated with oral doxycycline (100 mg every 12 hours) for a 7-day course. The patient demonstrated excellent adherence to the revised regimen and reported no adverse effects. Within 48 hours of doxycycline initiation, the patient’s fever resolved completely, followed by rapid improvement in headache and hearing symptoms. Full recovery of auditory function was confirmed by the completion of the therapeutic course. Notably, corticosteroid therapy was not administered, as the hearing loss and other systemic symptoms showed rapid and definitive improvement with doxycycline monotherapy.

### Follow-up and outcomes

During the 1-month and 3-month post-discharge follow-ups, the patient remained asymptomatic through comprehensive clinical assessments conducted by the treating neurologist. The evaluation confirmed complete resolution of initial symptoms including fever, headache, and auditory disturbances. Patient-reported outcomes indicated full restoration of hearing function and quality of life to pre-illness levels. While the patient declined repeat lumbar puncture due to the invasive nature of the procedure and complete clinical recovery, serial clinical monitoring demonstrated sustained wellness. The patient maintained perfect adherence to the 7-day doxycycline regimen and reported no adverse drug reactions. No unanticipated events or complications were documented during the treatment and follow-up period. Investigation of household contacts revealed no similar cases, supporting the arthropod-borne transmission hypothesis.

## Discussion

This case provides valuable insights into an unusual neurological manifestation of scrub typhus and the complex interplay between bacterial and viral pathogens. The transient, fluctuating nature of the hearing loss, coupled with its complete resolution following antibacterial therapy alone, points towards a novel, immune-mediated mechanism rather than direct bacterial damage to cochlear structures.

The most plausible explanation for the hearing loss in our patient is *O. tsutsugamushi*-induced vascular injury and subsequent inflammatory cascade within the inner ear. As an endothelial tropic pathogen, *O. tsutsugamushi* infection can trigger widespread vasculitis and perivascular inflammation (2). The inner ear is highly dependent on a stable blood supply maintained by the blood-labyrinth barrier. Compromise of cochlear microvasculature could disrupt endocochlear potential and ionic homeostasis, leading to fluctuating hearing function (4). The inner ear possesses a specialized immune environment, with resident macrophages and potential for adaptive immune responses against both pathogenss and self-antigens (5).

Recent studies have highlighted the role of upregulated pro-inflammatory cytokines, particularly the interleukins, in endothelial cells during systemic infections (6), which can recruit leukocytes and cause reversible inner ear dysfunction. The rapid normalization of hearing after doxycycline treatment suggests that the inflammatory process was truncated before irreversible hair cell damage occurred, aligning with the observed reversibility of cytokine-mediated auditory pathology in animal models (7).

The detection of EBV sequences in the Cerebrospinal Fluid(CSF), in conjunction with the serological profile indicating reactivation, introduces a critical dimension to the pathophysiology. While we cannot definitively prove EBV presence within the cochlea, its reactivation in the Central Nervous System(CNS) compartment is significant. *O. tsutsugamushi* is known to induce a state of transient immunosuppression, primarily through the upregulation of interleukin-10 and modulation of dendritic cell function (8). This immunosuppressed state may facilitate EBV reactivation from latency. Conversely, EBV itself employs sophisticated immun oevasion strategies (9). Once reactivated, EBV itself employs sophisticated immunoevasion strategies and can potentiate local inflammation (10). The concept of bystander activation of B-cells targeting shared antigens in the inner ear has been proposed in other viral infections (11). Thus, the coinfection may have created a synergistic pro-inflammatory environment within the CNS and potentially the inner ear, lowering the threshold for clinical manifestation of hearing loss. This hypothesis is supported by recent reports of autoimmune sensorineural hearing loss possibly triggered by neurocysticercosis (12).

The interferon (IFN) response represents a crucial interface for pathogen interaction. Both *O. tsutsugamushi* and EBV have evolved mechanisms to modulate IFN signaling (13, 14).The combined effect of these pathogens on the host’s antiviral defense could have altered the disease course. Unfortunately, we could not characterize the local cytokine profile in this case, leaving this intriguing mechanistic question for future investigation.

From a diagnostic perspective, this case underscores the paramount value of mNGS in identifying uncommon pathogens and cryptic coinfections (15). The technology was essential for directing appropriate therapy when conventional tests were non-diagnostic. The rapid clinical response to doxycycline alone suggests that the EBV detection represented an opportunistic reactivation secondary to the bacterial infection, not requiring specific antiviral therapy. This observation is critical for clinical decision-making in similar scenarios.

The main limitation of our study is the lack of acute-phase audiological evaluation to precisely characterize the hearing loss phenotype. Furthermore, while the EBV serology strongly suggests reactivation, more sophisticated tests on the CSF would be needed to confirm active viral replication.

## Patient perspective

The sudden hearing loss was the most frightening part of my illness. One moment I could hear, the next everything became muffled. I was terrified it might be permanent. The rapid improvement after starting the correct antibiotic was a huge relief. I am grateful to the medical team for their persistence. I hope my experience helps others with similar symptoms get the right diagnosis and treatment quickly.

## Conclusion

In conclusion, we describe a rare case of transient fluctuating hearing loss associated with *O. tsutsugamushi* and EBV coinfection. The findings expand the clinical spectrum of scrub typhus and highlight the potential for viral coinfection to modulate disease expression. The excellent response to doxycycline alone underscores that this auditory symptom can be a reversible, immune-mediated complication. Physicians in endemic regions should consider scrub typhus in the differential diagnosis of acute febrile illness with auditory symptoms, as prompt correct treatment can prevent permanent sequelae. Future research should explore the immunological interactions between *O. tsutsugamushi* and herpesviruses, and prospective studies with detailed audiometric testing are warranted to better characterize the evolution of hearing loss in these infections.

## Data Availability

The original contributions presented in the study are included in the article/Supplementary Material. Further inquiries can be directed to the corresponding author.
